# Application of Deep Learning Algorithms Based on the Multilayer Y0L0v8 Neural Network to Identify Fungal Keratitis

**DOI:** 10.17691/stm2024.16.4.01

**Published:** 2024-08-30

**Authors:** A.V. Sitnova, E.R. Valitov, S.N. Svetozarskiy

**Affiliations:** Clinical Resident, Department of Eye Diseases; The S. Fyodorov Eye Microsurgery Federal State Institution, 59a Beskudnikovsky Blvd., Moscow, 127486, Russia; Teacher, Computer Sciences Chair, Department of Big Data and Information Retrieval; National Research University Higher School of Economics, 20 Myasnitskaya St., Moscow, 101000, Russia; MD, PhD, Ophthalmologist; Privolzhsky District Medical Center of Federal Medico-Biologic Agency of Russia, 14 llyinskaya St., Nizhny Novgorod, 603000, Russia; Assistant, Department of Eye Diseases; Privolzhsky Research Medical University, 10/1 Minin and Pozharsky Square, Nizhny Novgorod, 603005, Russia

**Keywords:** deep learning, convolutional neural network, YOLOv8, corneal mycosis, keratitis diagnosis

## Abstract

**The aim of the study** is to develop a method for diagnosing fungal keratitis based on the analysis of photographs of the anterior segment of the eye using deep learning algorithms with subsequent evaluation of sensitivity and specificity of the method on a test data set in comparison with the results of practicing ophthalmologists.

**Materials and Methods:**

The study has included the stages of data acquisition, image pre-training and markup, selection of training approach and neural network architecture, training with input data augmentation, validation with hyperparameter correction, evaluation of algorithm performance on a test sample, and determination of sensitivity and specificity of fungal keratitis detection by practicing doctors. A total of 274 anterior segment images were used, including 130 photographs of the eyes affected by fungal keratitis and 144 photographs illustrating normal eyes, keratitis of other etiologies, and various anterior segment pathologies. Photographs taken after the treatment onset, illustrations of keratitis of mixed etiology and corneal perforation were excluded from the study. Images of the training sample were marked up using the VGG Image Annotator web application and then used to train the YOLOv8 convolutional neural network. Images from the test data set were also offered to practicing ophthalmologists to determine the diagnostic accuracy of fungal keratitis.

**Results:**

The sensitivity of the model was 56.0%, the specificity level reached 96.1%, and the proportion of correct answers of the algorithm was 76.5%. The accuracy of image recognition by practicing ophthalmologists was 50.0%, specificity — 41.7%, sensitivity — 57.7%.

**Conclusion:**

The study showed the high potential of deep learning algorithms in the diagnosis of fungal keratitis and its advantages in accuracy compared to expert judgment in the absence of metadata. The use of computer vision technologies may find application as a complementary diagnostic method in decision making in complex cases and in telemedicine care settings. Further research is required to compare the developed model with alternative approaches, to expand and standardize databases.

## Introduction

The abundance of visual information has evoked increased interest to the development and implementation of artificial intelligence into the sphere of eye health protection [1] including organization of telemedical care [2]. Machine learning-based medical technologies may act as a virtual physician’s assistant providing faster decision-making, improving diagnostic accuracy, and decreasing the risks of errors and data processing costs [3]. The last achievements in the field of deep learning algorithms, convolutional neural networks in particular, made it possible to reveal diseases using computer vision technologies trained on large sets of medical images [4, 5].

The greatest success of machine learning in ophthalmology has been achieved in automated diagnosis of diabetic retinopathy, age-related macular degeneration, glaucoma, and cataract [1, 6]. But the capabilities of artificial intelligence for early detection of corneal diseases remain yet insufficiently studied. It is connected with the fact that image acquisition of the anterior eye segment is less unified in comparison with photoregistration of the eye fundus due to the diversity of structures and imaging methods [7].

Corneal opacity is the cause of bilateral blindness and distinct vision reduction in 5.5 million people in the world and the cause of unilateral vision loss in 6.2 million people [8]. A significant proportion of corneal opacities is connected with previous infectious keratitis, the incidence of which ranges from 6.6 and 27.6 cases per 100,000 population annually in various regions of Australia and the USA, respectively, and up to 113 and 799 cases per 100,000 population in India and Nepal, respectively [9].

The share of fungal lesions in the structure of infectious keratitis morbidity is increasing each year due to a wide and uncontrolled use of eye drops containing antibiotics and steroids [10]. The disease prevails mainly in tropical and subtropical regions, where its rate reaches 81.5% of all laboratory validated corneal infections [11, 12]. The burden of fungal keratitis falls on patients and society as it causes long-term disability and invalidization, prolonged and expensive treatment and rehabilitation, lower quality of life, and limited social functioning due to the loss of visual functions [13]. Fungal keratitis has been defined as “a social and economic catastrophe” in view of its high social significance [14].

Fungal infection is known by its aggressive course and resistance of the causative agent to anti-fungal drugs, which is connected with specific pathogenesis and biofilm formation [10]. Late beginning of specific treatment is associated with complications, emergency operations, and vision loss. Early detection of fungal keratitis is hindered by insufficient accuracy of clinical diagnosis amounting to 33-80% [15], false-positive results of cultural microbiological investigation methods reaching 37-41% [11, 16], and limited availability of new highly informative methods [10]. All this determines the importance of searching for new methods of early diagnosis of the disease [9, 10, 17], meeting the requirements of rapid performance, high accuracy, independence on the clinical experience of the specialists, and wide availability. These requirements may be met by the diagnostic systems based on the technologies of artificial intelligence, where multilayer neural networks are used — deep learning. At the same time, only single studies are devoted to the analysis of artificial intelligence capabilities in the field of anterior segment recognition.

**The aim of the study** is to develop a method for diagnosing fungal keratitis based on the analysis of photographs of the anterior segment of the eye using deep learning algorithms with subsequent evaluation of sensitivity and specificity on a test data set in comparison with the results of practicing ophthalmologists.

## Materials and Methods

The study included the following stages: data acquisition, image pre-training and markup, selection of a training approach and neural network architecture, training of neural network, validation with hyperparameter correction, evaluation of algorithm performance on a test sample, determining sensitivity and specificity of fungal keratitis detection by practicing doctors. The study complies with the Declaration of Helsinki (2013).

Data were acquired retrospectively from the base of photographs of the anterior eye segment taken with Visucam 500 fundus camera (Carl Zeiss, Germany) at the Department of Ophthalmology of Privolzhsky District Medical Center of Federal Medico-Biologic Agency of Russia in the period from 2015 to 2022. The obtained data set was supplemented from PubMed Central and Retina Image Bank. The main criteria were adequate image quality meeting the parameters of focusing, centration, brightness, accessibility of cornea for viewing (eyelid coverage should not exceed 1/3), and reliable information on patient diagnosis.

At the stage of data preparation, images were divided into classes, including 130 photographs of the eyes affected by fungal keratitis and 144 photographs illustrating physiological norm and other kinds of pathology of the anterior segment, including cataract, corneal dystrophy, pterygium, keratitis of viral and bacterial etiology.

In connection with the difficulties of standardization of anterior segment images, criteria for complex quality evaluation of such images were proposed in 2023 for their inclusion into data sets when developing artificial intelligence systems [18]. These criteria were used by us for standardization of the data set and improvement of the study reliability. Each image was assessed in scores based on the parameters such as resolution, format, distinguishing features of the pathology of interest, image source, anatomic integrity, and information about the patient (Table 1).

**T a b l e 1:** Image standardization parameters and their assessment [18]

Parameter	Score	Criteria
Image resolution	10	Above average
	5	Average/below average, but all structures are discernible
	0	Below average, the structures are difficult to distinguish
Image format	10	Image format matches the format of the data set
	0	Image format does not match the format of the data set
Distinguishing features	10	There are characteristic features of the disease visible distinctly in the photograph
	0	There are no characteristic features or they are not visible in the photograph
The source of the image	10	Image from the original source, from the known patient
	5	Original source is unknown, but there is exact diagnosis and characteristic features of the disease
	0	Unauthentic image, the diagnosis is doubtful or unknown
Anatomical integrity	10	Fully preserved
	0	Not preserved
Ophthalmic history	10	The history and diagnosis are known
	5	The history is unknown, but there is exact diagnosis
	0	No data about the patient or diagnosis

Images with a “0” rating for least one parameter were excluded from the database. Preference was given to the photographs from the archive of our own fundus camera having the score about 10 on all points.

The presence of the verified fungal corneal lesion of any size and location was the criterion of inclusion in the group of fungal keratitis. At the same time, photographs taken after the treatment onset and those illustrating keratitis of mixed etiology and corneal perforation were excluded. The second group comprised images with physiological norm and various pathologies of the organ of sight visualized in the photographs of the anterior eye segment (diseases of cornea, iris, lens), which might be mistaken for fungal damage of the cornea. For neural network training, photographs from each group were randomly divided into the training, validation, and test samples (Table 2). All images were converted to the same format of 320x320 pixels and anonymized.

**T a b l e 2:** Distribution of data in the samples

Pathology	Total number of images	Training sample	Validation sample	Test sample
Fungal keratitis	130	84	20	26
Other pathologies + physiological norm	144	98	20	26

**Figure 1.**
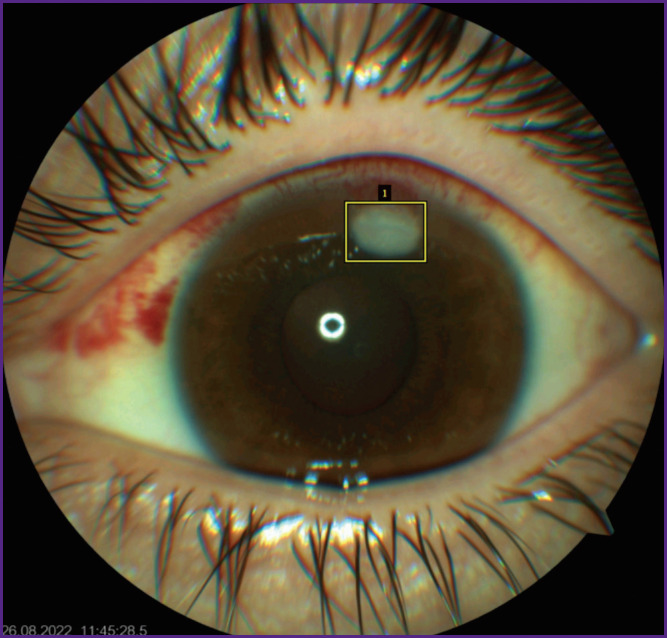
Manual mark-up of the anterior eye segment photograph of the patient with fungal keratitis using the method of 2D-bounding boxes

At the markup stage, the region of interest was isolated in all images of the training sample using vector 2D bounding boxes with the help of VGG Image Annotator, a manual annotation software (Figure 1). In the marking process we followed some principles: the zones of interest were isolated with boxes, several small closely located foci were united by a single frame, the marking boundaries were set as close to the focus margins as possible. The frame was positioned so that the eyelid edges and light reflexes were beyond its limits.

The choice of approaches to learning and creation of the architecture of machine learning algorithms were determined by the goal set: to differentiate the corneal affection by a fungal agent from other lesions. From the point of view of technical terminology, this implies solving the task of classification of images and detection of objects. In this connection, the method of supervised learning to train the multilayer neural network has been chosen. The YOLOv8 (You Only Look Once) algorithm for real-time classification and object detection was selected from the variety of convolutional neural networks. The YOLOv8 model demonstrates higher speed and accuracy in different fields of computer vision relative to the models of the previous generations [19]. In contrast to other two-level convolutional neural networks, YOLOv8 evaluates simultaneously all selected image regions making the learning process significantly faster [20].

To train the neural network, we used 182 marked-up images of the training sample. The learning principles may be described as a number of successive stages. First, the image is fed to the input of the neural network, where its analysis and formation of values take place; these values are used by the network to compare the regions of interest with the areas of the input image. Further, regions, in which objects subject to recognition, are selected [21]. In this way, a test model capable of analyzing a set of features and classifying objects is created. The algorithm checks its result with the specified one and corrects the model in compliance with the “correct” answer. This process is repeated many times until the algorithm can classify the objects correctly. Additionally, various augmentations are applied at each iteration, i.e. insignificant changes in the object (for example, reduction or zoom-in). This action allows to expand the training sample artificially, making the model more resistant to the photographs of different quality and angle.

Training of the model was finished, when improvements were not registered any more permitting us to avoid overfitting.

At the next stage, the accuracy of the trained model was determined using an unknown, completely unique, validation sample of 40 images, which allowed for manual correction of the external configuration variables, or hyperparamters. Once the satisfactory values of model sensitivity were achieved, the test sample was fed to the input, having no repetitions with the training and validation datasets, to determine indicators of efficiency of fungal keratitis diagnosis. The study was performed using NVIDIA GTX TITAN Xp Pascal hardware with 12 GB of memory necessary for image recognition and processing.

Four ophthalmologists with over 7-year experience have been proposed to diagnose fungal keratitis by the photographs of the test sample to compare sensitivity and specificity of the trained neural network and practicing specialists. Each expert had to detect images with fungal corneal lesion without any additional information about patients, which made the task more difficult [22].

**Statistical data analysis** included calculation of indicators of sensitivity, specificity, accuracy, precision, and recall as well as building precision-recall curves for evaluation of the fungal keratitis detection quality in the images of the anterior eye segment using the designed model. Precision-recall curves are similar to ROC for visualizing the results of machine learning algorithms. The indicators of model performance and practicing ophthalmologists were compared using *χ^2^* criterion; the accepted level of significance was 0.05.

## Results

**Figure 2.**
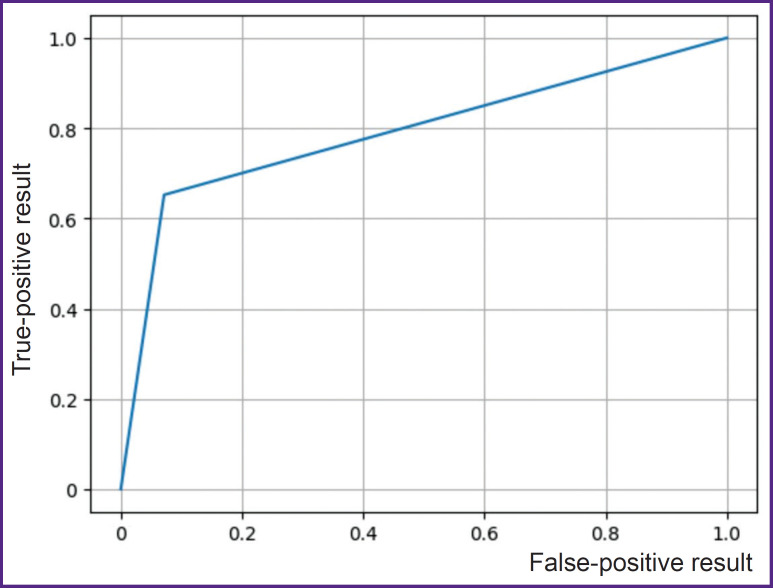
Precision-recall curve characterizing the quality of fungal keratitis detection in the anterior segment images using the designed model

The process of training the YOLOv8 neural network included 118 iterations and lasted for 413 min. The share of correct answers on the test sample was 76.5%, while error probability 23.5% (Figure 2). The results of testing showed model sensitivity equal to 56%, specificity — 96% (Table 3). Classification of 52 photos by the experienced ophthalmologists demonstrated that the average value of correct answers (Acc) was 50%, specificity — 41.7%, sensitivity — 57.7%. The accuracy and specificity of the developed algorithm exceeded significantly the results of the expert assessment (p<0.05) (see Table 3).

**T a b l e 3:** Results of image classification by the designed model and practicing specialists

Indicators	Model	Specialists	P
Accuracy (%)	76.5	50.0	0.004
Sensitivity (%)	56.0	57.7	1.0
Specificity (%)	96.1	41.7	<0.001
Precision	0.93	—	—
Recall	0.56	—	—

**Figure 3.**
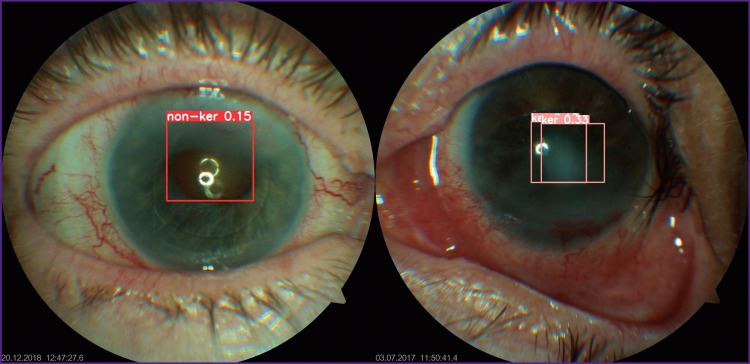
Results of dichotomic classification of images and detection of the test sample objects using the designed model The label ‘ker’ corresponds to the identification of fungal keratitis signs by the algorithm with indication of the probability of this event; ‘non-ker’ denotes absence of the signs of fungal keratitis with the probability of this outcome

As the result of the test sample evaluation by the algorithm we have obtained the marked-up images with the marks corresponding to the model’ assumption as to whether the isolated pathology is fungal keratitis (Figure 3). It was established that the model marked up images adequately in 76.5% of cases.

Thus, the present investigation has demonstrated the high performance of the machine learning algorithms, developed on the basis of the convolutional multilayer YOLOv8 neural network in diagnosis of fungal keratitis in the photographs of the anterior eye segment compared to the results of the practicing ophthalmologists. The originality of the approach was in combining the tasks of image classification with detection of pathological objects and including non-inflammatory diseases of the anterior segment in the data set, which brought the modeled situation as close to the conditions of telemedical monitoring as possible.

## Discussion

Clinical data, including the past history and objective picture detected by microscopy, underlie the differential diagnosis of keratitis of various etiologies. The most complex task for verification is a fungal lesion, in which the accuracy of expert evaluation by practicing ophthalmologists is characterized by a wide range of values. For instance, the share of correct answers in differentiation of fungal and bacterial keratitis by the photographs of the anterior segment is 49-67% [22–24], which correlates with the values obtained by us (50%). The relatively low indicators of efficiency may be connected with the fact that the doctors engaged in our study were not narrow specialists in infectious eye pathology, but at the same time, they had at least 5-year experience in corneal transplantation.

The comparison of algorithm accuracy values with the results of practicing specialists is an important component of investigations devoted to the development of computer vision technologies. For example, in the study performed by Kuo et al. [24], the algorithm with a similar design based on the DenseNet architecture, demonstrated higher sensitivity and lower specificity relative to practicing ophthalmologists. Expert diagnosis of infectious keratitis, as shown in the work by Hu et al. [25], was also inferior to the suggested methods of machine learning.

The diagnostic accuracy of deep learning algorithms depends primarily on the amount and quality of the data used, and there is a direct correlation between the amount of data and the accuracy of the results [6]. Soleimani et al. [26] have managed to compile a unique database of 9329 photographs of 977 patients taken in the process of biomicroscopy, which allowed them to achieve 84% accuracy in differentiating between bacterial and fungal keratitis and 77.5% in identifying the type of fungi (filamentous or yeast pathogens). Koyama et al. [27] were solving the tasks of etiological diagnosis using the ResNet50-based algorithm, which provided high accuracy in diagnosing acanthamoeba (96.7%), bacterial (77.6%), and fungal (84.2%) corneal lesions, while the share of correct answers for herpes simplex virus reached 91.7%.

In order to improve the method developed by us, it is necessary to further enlarge the sample volume by increasing the number of fungal keratitis images as well as images of corneal lesions of a different etiology and other diseases of the anterior eye segment, which will enhance the algorithm efficiency. The ways of solving this problem include involvement of new research centers possessing unique bases of ophthalmological images. An example of such collaboration is the work of Tiwari et al. [28], where replenishment of the photograph base was done by uniting the data sets stored in the repository of clinical trials under the programs “Steroids for Corneal Ulcers Trial (SCUT)” and “Mycotic Ulcer Treatment Trial (MUTT)” as well as images from the local archive of Stanford University.

In the future, the indicators achieved by us may be improved not only by enlarging a sample volume but by upgrading the data quality, improving criteria of photograph selection, and a more detailed working out of neural network parameters. This is hindered by the criteria of inclusion and exclusion of objects from the sample, since it happens rather often that patients visit ophthalmologist after unsuccessful attempts of self-treatment or at the late stages of the disease, when ulcerative changes, cornea perforation, or joining a secondary infection may be observed. There is also a need in unification of image parameters. For instance, the uncorrected brightness of the photographs may cause the low values of the algorithm correct answers. When selecting the data, obviously bright and too dark images were excluded from the sample, however, the exact determination of the optimal range of brightness is a serious scientific task [29]. Hanif et al. [30] have analyzed the effect of quality of anterior segment photographs on the neural network performance and established that presence of the light reflexes and getting eyelid and lash margins into the frame influenced significantly final diagnostic indicators. Marking up the images elevated the accuracy by 16% [31], while inclusion of the automatic image segmentation stage into the algorithm might increase the performance by 7% [32].

The choice of a neural network architecture also influence the system performance, since its characteristics are selected depending on the type of information and the tasks of the investigation. A number of works devoted to the analysis of ophthalmological images were successfully implemented based on the neural networks such as ResNet50 and FasterCNN [31]. In the study [33], devoted to the classification of bacterial and fungal keratitis, the ResNet50-based model has shown 80% sensitivity and 70% specificity, exceeding a bit our values. However, it may be accounted for by the sample size, exclusion of some types of corneal and anterior segment pathology, and the use of the pretrained neural network. When comparing the diagnostic accuracy of several neural networks, the accuracy of diagnosing fungal keratitis varied from 26 to 66%, whereas for bacterial keratitis the share of correct answers was about 79.6% [34]. In contrast to a fungal lesion, the comparison of algorithm performance on the basis of ResNet50, ResNeXt50, DenseNet121, SE-ResNet50, EfficientNets BO, B1, B2, and B3 neural networks in the recognition of bacterial keratitis did not show statistically significant differences [35]. Taking into account the lack of the studies using a new generation of YOLOv8 neural networks for detection of fungal keratitis, we consider reasonable further exploration of the capabilities of this architecture as compared to other convolutional neural networks used to analyze a large volume of images.

To improve the algorithms of deep learning, it is important to understand cases in which false-positive and false-negative results appear. Analyzing the causes of errors, Gu et al. [36] have established that the model falsely diagnosed cataract in patients with affected central zone of the cornea due to the localization of the lesion in the iris area. Complicated cataract was identified by the algorithm as infectious keratitis, whereas non-infectious keratitis was recognized as infectious by both the neuronal network and ophthalmologists.

Considerable success in diagnosing keratitis of various etiologies using deep learning methods was achieved in the analysis of cornea images obtained during confocal microscopy; fungal keratitis was identified with 97% accuracy and 96% specificity [37].

However, a limited availability of confocal microscopy encourages further research in the field of recognition of anterior segment photographs [10], which is especially important for data exchange in the process of telemedical consultations. All this proves the complexity of identifying fungal keratitis and confirms the need to optimize algorithm performance and enrich data sets.

Apart from technical difficulties occurring in the process of model training, researchers and ophthalmologists face a question of how wide and how soon these technologies may be applied. The methodology of our study suggested the recognition of anterior segment photographs without metadata. In this connection, the comparison of the results obtained by the experts and the designed algorithm cannot be fully extrapolated to the conditions of real clinical practice, since even under the conditions of teleconsultations the specialist possesses additional information from the patient’s medical history valuable for diagnosing. The technologies of computer vision make it possible to classify images in the categories, which has a great potential for practical medicine and the development of healthcare as a whole. Digital medicine was called a leading direction for improvement of public health according to the strategy of developing manufacturing industry of the Russian Federation up to 2035 [38]. Implementation of artificial intelligence technologies will make it possible to perform a complex screening of many diseases including fungal keratitis. However, it involves a number of challenges, many of which remain unsolved so far. For example, ethical problems of using patient data, provision of information safety, and legal liability are being discussed. A technical limitation for the technology development is the lack of unified standards for data acquisition. The researchers in various clinics are using their own original principles of data collection and processing, which results in low reproducibility of the results even in case of similar technical support. Both ethical and technical aspects should be regulated, which requires improvement of the legislative base in the sphere of biomedical artificial intelligence.

The advantages of the present study consist in the sufficient volume of images with rare ophthalmological pathologies, marking up of all photographs in the training sample, and the comparative character of the work. It is for the first time that the solution of the task of automated fungal keratitis diagnosis in combination with the detection of pathological objects in the photographs of the anterior eye segment using the YOLOv8 neural network is presented in the scientific literature.

## Conclusion

Visualization of the anterior eye segment structures underlies the diagnosis of corneal diseases, however, interpretation of the data obtained depends on the experience of ophthalmologists, which may influence the terms of rendering assistance and administration of adequate treatment. The clinical approach alone cannot always justify its simplicity, including cases with fungal keratitis, which requires prompt and early diagnosis. Automated classification of medical images has a significant potential in solving these problems, since the application of artificial intelligence promotes the reduction of healthcare specialist workload and improves the efficiency of the screening methods.

Our study presents the method of diagnosing fungal keratitis using the convolutional YOLOv8 neuronal network for automated classification and detection of pathological objects in the photographs of the anterior eye segment. The share of the correct answers of the algorithm was 76.5%, sensitivity and specificity — 57.7 and 96.1%, respectively, which was not inferior to the average indicators of the practicing ophthalmologists.

The perspective tasks for further investigations will include the acquisition of a larger volume of data and their thorough processing, unification of technical parameters, improvement of deep learning algorithms for better performance, assessment of the capabilities of the method for differential diagnosis of various types of corneal pathology.
